# Macrolide antibiotics block autophagy flux and sensitize to bortezomib via endoplasmic reticulum stress-mediated CHOP induction in myeloma cells

**DOI:** 10.3892/ijo.2013.1870

**Published:** 2013-03-28

**Authors:** SHOTA MORIYA, XIAO-FANG CHE, SEIICHIRO KOMATSU, AKIHISA ABE, TOMOHIRO KAWAGUCHI, AKIHIKO GOTOH, MASATO INAZU, AKIO TOMODA, KEISUKE MIYAZAWA

**Affiliations:** 1 Departments of Biochemistry, Tokyo Medical University, Tokyo, Japan; 2 Breast Oncology, Tokyo Medical University, Tokyo, Japan; 3 Hematology, Tokyo Medical University, Tokyo, Japan; 4 Institute of Medical Science, Tokyo Medical University, Tokyo, Japan

**Keywords:** bortezomib, clarithromycin, myeloma, ER-stress, autophagy, CHOP, macrolide antibiotics

## Abstract

The specific 26S proteasome inhibitor bortezomib (BZ) potently induces autophagy, endoplasmic reticulum (ER) stress and apoptosis in multiple myeloma (MM) cell lines (U266, IM-9 and RPMI8226). The macrolide antibiotics including concanamycin A, erythromycin (EM), clarithromycin (CAM) and azithromycin (AZM) all blocked autophagy flux, as assessed by intracellular accumulation of LC3B-II and p62. Combined treatment of BZ and CAM or AZM enhanced cytotoxicity in MM cell lines, although treatment with either CAM or AZM alone exhibited almost no cytotoxicity. This combination also substantially enhanced aggresome formation, intracellular ubiquitinated proteins and induced the proapoptotic transcription factor CHOP (CADD153). Expression levels of the proapoptotic genes transcriptionally regulated by CHOP (BIM, BAX, DR5 and TRB3) were all enhanced by combined treatment with BZ plus CAM, compared with treatment with each reagent alone. Like the MM cell lines, the CHOP^+/+^ murine embryonic fibroblast (MEF) cell line exhibited enhanced cytotoxicity and upregulation of CHOP and its transcriptional targets with a combination of BZ and one of the macrolides. In contrast, CHOP^−/−^ MEF cells exhibited resistance against BZ and almost completely canceled enhanced cytotoxicity with a combination of BZ and a macrolide. These data suggest that ER stress-mediated CHOP induction is involved in pronounced cytotoxicity. Simultaneously targeting two major intracellular protein degradation systems such as the ubiquitin-proteasome system by BZ and the autophagy-lysosome system by a macrolide antibiotic enhances ER stress-mediated apoptosis in MM cells. This result suggests the therapeutic possibility of using a macrolide antibiotic with a proteasome inhibitor for MM therapy.

## Introduction

Proteasome inhibition has emerged as an important therapeutic strategy for multiple myeloma (MM). For many years, the combination of oral melpharan and prednisolone (MP) has been a conventional treatment for MM patients. Bortezomib (Velcade, BZ) became the first-in-class proteasome inhibitor to be introduced into clinic treatment 10 years ago and is now predominantly used in combination regimens such as VMP (consisting of BZ and MP) and VTD (BZ plus thalidomide and dexamethasone) in MM patients (1–4). Although regimen involving BZ has contributed to substantial improvement in survival in MM compared to conventional MP therapy, MM is still an incurable neoplasm with median survival ranging from 3 to 6 years (3–5). Therefore, further therapeutic improvement remains a crucial issue.

Constitutive nuclear factor (NF)-κB activity in MM cells mediates survival, as well as resistance to chemotherapy and radiotherapy, by inducing the expression of anti-apoptotic proteins, adhesion molecules and autocrine growth factors such as interleukin-6 (6,7). Since IκBα is a substrate of the proteasome, the initial rationale for using BZ in MM was to inhibit NF-κB (8,9). However, recent reports demonstrated instead that BZ activates the canonical pathway of NF-κB in MM and lymphoma cells; therefore, the inhibition of NF-κB activity is not involved in the therapeutic effect of BZ (10–14). It was reported that BZ-induced calpain-dependent IκBα degradation facilitated p65 nuclear translocation and increased NF-κB activity (12). It was also demonstrated that BZ treatment promoted IκBα phosphorylation, ubiquitination and degradation via the autophagy-lysosome degradation system, resulting in increased NF-κB nuclear translocation and transcription activity in diffuse large B-cell lymphoma cells. Therefore, blocking autophagy with chloroquine prevented BZ-induced NF-κB activation by reducing IκBα degradation and enhanced BZ-induced killing of lymphoma cells (13). Immunohistochemistry using anti-p65 antibody in MM cells derived from 60 samples of MM patients confirmed that NF-κB was almost exclusively expressed in the cytoplasm, which indicated its inactive form. In addition, BZ exhibited consistent antitumor activity against MM cells, regardless of NF-κB localization (14). All these data suggest the existence of another molecular mechanism underlying BZ-mediated cytotoxicity.

Increasing lines of evidence indicate that inhibition of the 26S proteasome by BZ leads to the accumulation of misfolded proteins in the endoplasmic reticulum (ER) (10,15–19). This results in ER stress followed by a coordinated cellular response known as unfolded protein response (UPR). Since MM is characterized by the uncontrolled cell growth of monoclonal antibody-producing plasma cells, large quantities of unfolded or misfolded immunoglobulin production itself triggers ER stress. ER stress is caused by an imbalance between the amount of unfolded or misfolded protein in the ER lumen and the capacity of the ER machinery to refold these proteins (20). The main functions of UPR are to reduce the amount of protein that enters the ER by suppuration of translational rate and to increase the folding capacity of the ER via translational activation of chaperon proteins. Additionally, if proteins cannot be folded correctly in the ER, they are retrotranslocated to the cytoplasm for degradation via the ubiquitin-proteasome pathway, a process termed ER-associated degradation (ERAD) for adaptation. However, if these strategies for adaptation fail, apoptosis is triggered with the induction of a proapoptotic transcription factor CHOP and with the IRE1 involved in signaling via caspase-12 (20–22). Thus, therapeutic manipulation of this pathway using BZ and other reagents might interfere with the ability to deal with high protein loads, cellular stress and might result in induction of MM cell death (10,18).

Macroautophagy (hereafter, autophagy) is a highly conserved cellular process in eukaryotes. Intracellular proteins and organelles including the ER are engulfed in a double-membrane vesicle called an autophagosome and are delivered to lysosomes for degradation by lysosomal hydrolases (23,24). Autophagy has been regarded as a bulk non-selective degradation system for long-lived proteins and organelles, in contrast to the specific degradation of polyubiquitinated short-lived proteins by proteasome. However, recent reports revealed the selective degradation pathway of ubiquitinated protein through autophagy via docking proteins such as p62 and the related protein NBR1, having both a microtubule-associated protein 1 light chain 3 (LC3)-interacting region and a ubiquitin-associated domain (25,26). LC3 is essential for autophagy and is associated with autophagosome membranes after processing (27). By binding ubiquitin via their C-terminal ubiquitin-associated domains, p62-mediated degradation of ubiquitinated cargo occurs by selective autophagy. Thus the two major intracellular degradation systems are directly linked (25,26). We have reported on the inhibition of autophagy using the autophagy inhibitor bafilomycin A_1_ enhanced BZ-induced apoptosis by burdening ER stress in MM cell lines (10). It was also reported that macrolide antibiotics such as clarithromycin (CAM) and azithromycin (AZM) attenuated or blocked autophagy flux, probably mediated through inhibiting the lysosomal function (28,29).

We therefore investigated whether simultaneous inhibition of protein degradation systems such as the ubiquitin-proteasome system by BZ and the autophagy-lysosome system by a macrolide antibiotic enhances the loading of ER-stress and ER-stress-mediated CHOP induction, followed by transcriptional activation for proapoptotic genes. In the present study, we clearly demonstrate that the combination of BZ and a macrolide such as AZM, CAM, or erythromycin (EM) enhances ER stress-mediated cytotoxicity via transcriptional activation of CHOP. Our data suggest that BZ and a macrolide antibiotic is a promising combination for MM therapy.

## Materials and methods

### Reagents

BZ was purchased from Toronto Research Chemical Inc. (North York, Ontario, Canada). BZ was dissolved in dimethyl sulfoxide (DMSO) at a concentration of 1 mM as a stock solution. CAM, bafilomycin A_1_ and concanamycin A were purchased from Wako Pure Chemical Industries (Osaka, Japan), EM was purchased from Sigma-Aldrich (St. Louis, MO) and AZM was purchased from Tokyo Chemical Industry (Tokyo, Japan). Bafilomycin A_1_, concanamycin A and AZM were dissolved in DMSO to make stock solutions of 10 *μ*M, 10 *μ*M, and 10 mg/ml, respectively. CAM and EM were dissolved in ethanol to make stock solutions of 5 and 10 mg/ml. E-64d and pepstatin A, which are inhibitors of lysosomal proteases, were purchased from Peptide Institute (Osaka, Japan).

### Cell lines and culture conditions

For this study, MM cell lines IM-9, U266 and RPMI8226 cells were obtained from the American Type Culture Collection (ATCC) (Manassas, VA). A CHOP^−/−^ MEF cell line (CHOP-KO-DR) established from a 13.5-day-old CHOP^−/−^ mouse embryo by SV-40 immortalization and a CHOP^+/+^ MEF cell line (DR-wild-type) established by SV-40 immortalization as a control cell line for CHOP-KO-DR were also obtained from ATCC. IM-9, U266 and RPMI8226 cells were maintained in continuous culture in RPMI-1640 medium (Gibco, Grand Island, NY) supplemented with 10% FBS (PAA Laboratories, Austria), 2 mM L-glutamine, penicillin (100 U/ml) and streptomycin (100 *μ*g/ml) (Wako) CHOP-KO-DR and DR-wild-type cells were maintained in Dulbecco’s modified Eagle’s medium (Sigma) supplemented with 10% FBS, penicillin (100 U/ml) and streptomycin (100 *μ*g/ml). All cell lines were cultured in a humidified incubator containing 5% CO_2_ and 95% air at 37°C.

### Assessment of the viable number of cells among cultured cells

The number of viable cells was assessed by CellTiter Blue, a cell viability assay kit (Promega Co., Madison, WI), with fluorescence measurements at 570 nm for excitation and 590 nm for fluorescence emission.

### Immunoblotting

Immunoblotting was performed as previously described (30). In brief, cells were lysed with RIPA lysis buffer (Nacalai Tesque, Kyoto, Japan) containing 1 mM PMSF, 0.15 U/ml aprotinin, 10 mM EDTA, 10 mg/ml sodium fluoride and 2 mM sodium orthovanadate. Cellular proteins were quantified using a DC Protein Assay kit of Bio-Rad (Richmond, CA). Equal amounts of proteins were loaded onto the gels, separated by SDS-PAGE and transferred onto Immobilon-P membrane (Millipore Corp., Bedford, MA). The membranes were probed with first antibodies (Abs) such as anti-LC3B antibody (Ab) (Novus Biological, Inc., Littleton, CO), anti-p62 monoclonal (m) Ab (sequestsome-1), anti-ubiquitin mAb and anti-GAPDH mAb (Santa Cruz, CA), anti-cleaved-caspase-3 Ab, anti-PARP Ab, anti-CHOP mAb (Cell Signaling Technology, MA). Immunoreactive proteins were detected with horseradish peroxidase-conjugated second Abs and an enhanced chemiluminescence reagent (ECL) (Millipore). Densitometry was performed using a Molecular Imager, ChemiDoc XRS System (Bio-Rad).

### Gene expression analysis

Total RNA was isolated from cell pellets using Isogen (Nippon Gene, Tokyo, Japan) and genomic DNA was removed using RQ1 RNase-Free DNase (Promega) at 37°C for 30 min, followed by extraction with phenol chloroform and ethanol precipitation. Reverse-transcription using a PrimeScript RT Master Mix (Takara Bio Inc. Ohtsu, Japan) was performed according to the manufacturer’s instructions. Real-time PCR was performed on 3 ng of cDNA using validated SYBR Green gene expression assays for human and mouse ER-stess related genes (*CHOP, BAX, BIM, DR5, GADD34* and *TRB3*) in combination with SYBR Premix Ex Taq II Tli RNase H Plus (Takara Bio Inc.). The sequences of primers are listed in Table I. Quantitative real-time PCR was performed in duplicates in a Thermal Cycler Dice Real-Time System TP800 (Takara) under the following conditions: initial cDNA denaturation at 95°C for 30 sec, followed by 45 cycles of the sequence of denaturation at 95°C for 5 sec and simultaneous annealing and extension at 60°C for 30 sec. The data were analyzed using Thermal Cycler Dice Real-Time System Software (Takara) and the comparative *C_t_* method (2^−ΔΔ*C*^_t_) was used for relative quantification of gene expression. The data of real-time PCR products were standardized to *GAPDH* as an internal control. To confirm the specific amplification of target genes, each gene product was further separated by 1.5% agarose gel after real-time PCR to detect a single band at the theoretical product size, as well as analysis of the dissociation curve for detecting a single peak.

### Assessment of aggresome formation

Assessment of aggresome formation was performed using a ProteoStat^®^ Aggresome Detection kit according to the manufacturer’s instructions (Enzo Life Sciences, Farmingdale, NY) (31). Cells were fixed with 4% paraformaldehyde, permeabilized with 0.5% Triton X-100 and incubated with ProteoStat aggresome dye. Aggresome was analyzed by flow cytometry using a Partec PAS I Flow Cytometer (Partec, Münster, Germany) with a 488-nm laser with fluorescence detection in the FL3 channel. After staining with ProteoStat aggresome dye, cells were further stained with 4′,6-diamidino-2-phenylindole (DAPI) and cell suspensions were sedimented and fixed on slide glasses using Shandon Cytospin III (Shandon Southern Products Ltd., Cheshire, UK) to make slide glass preparations. Analysis by fluorescence microcopy was performed using a Texas Red filter for imaging the cell aggresome signal and a DAPI filter for imaging the nuclear signal using a digital microscope BZ-9000 (Keyence Co., Osaka, Japan).

### Assessment of apoptosis

Cells were stained with Annexin V and propidium iodide (PI) using an Annexin V-FITC Apoptosis Detection kit (Wako) according to the manufacturer’s protocol. Fluorescent intensities were detected by flow cytometry using a Partec PAS I flow cytometer (Partec). Annexin V-FITC binding was monitored using an FITC signal detector (FL1, 520 nm) and PI staining was monitored phycoerythrin emission signal detector (FL3, 590–650 nm). We also performed morphological observation for assessment of apoptosis. Cell suspensions were sedimented and fixed on slide glasses using Shandon Cytospin III (Shandon Southern Products Ltd.); preparations were then stained with May-Grünwald-Giemsa and examined using a digital microscope BZ-9000 (Keyence Co.).

### Statistics

All data are given as the mean ± SD. Statistical analysis was performed by using Mann-Whitney’s U test (two-tailed).

## Results

### Apoptosis and autophagy induction after treatment with BZ in MM cell lines

**Figure 1:**
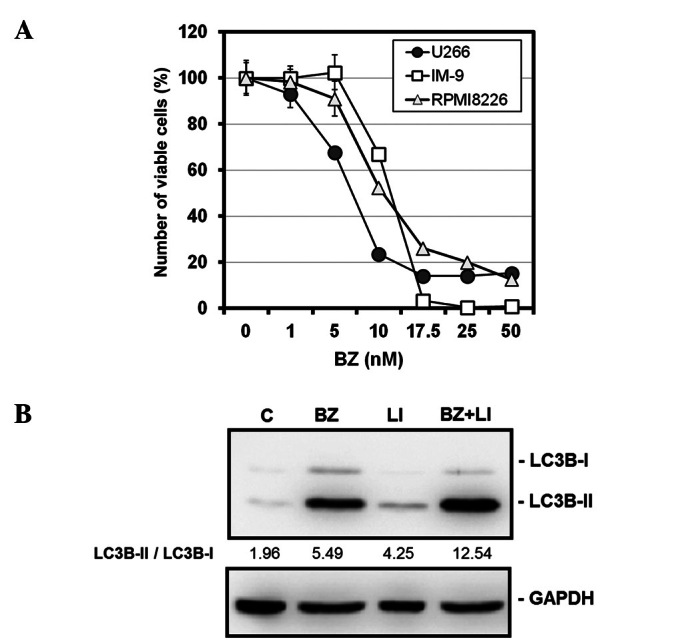
Cell growth inhibition and autophagy induction in MM cell lines after treatment with BZ. (A) U266, IM-9 and RPMI8226 cells were treated with BZ at various concentrations for 48 h. The number of viable cells was assessed by CellTiter Blue as described in Materials and methods. (B) U266 cells were cultured with or without BZ (10 nM) in the presence or absence of lysosomal inhibitors (LI), E-64d (10 *μ*g/ml) and pepstatin A (10 *μ*g/ml) for 48 h. Cellular proteins were separated by 15% SDS-PAGE and immunoblotted with anti-LC3B Ab. Immunoblotting with anti-GAPDH mAb was performed as an internal control. The numbers indicate the ratio of LC3B-II/LC3B-I as determined by densitometry.

BZ induced cell growth inhibition in a dose-dependent manner in all three MM cell lines tested. IC_50_ (50% inhibitory concentrations) of each cell line was 7.2 nM for U266, 10.5 nM for RPMI8226 and 12.2 nM for IM-9, respectively (Fig. 1A). Morphological features and immunoblotting with anti-cleaved caspase-3 and anti-PARP Abs all revealed apoptosis induction after treatment with BZ, as previously reported elsewhere (10). Immunoblottings with anti-LC3B and anti-p62 Abs demonstrated that treatment with myeloma cells with BZ resulted in increased expression ratios of LC3B-II to LC3B-I, along with decreased expression levels of p62 (10). Combined treatment with BZ and lysosomal inhibitors such as pepstatin A and E64d further increased the ratio of LC3II-B to LC3B-I, compared with those after treatment with either BZ or lysosomal inhibitors alone in U266 cells (Fig. 1B). This result indicated that increased ratios of LC3B-II to LC3B-I in response to BZ are due to autophagy induction rather than blocking autophagic flux as previously reported (27,32).

### Macrolide antibiotics blocked autophagy flux and sensitized to BZ in MM cells

**Figure 2:**
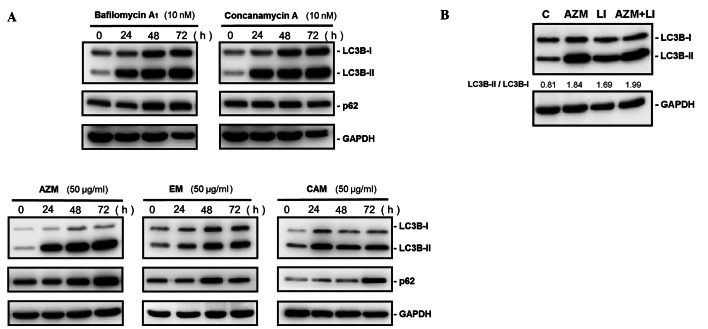
Immunoblottings with anti-LC3B Ab and anti-p62 Ab after U266 cells were treated with various macrolide antibiotics. (A) U266 cells were treated with bafilomycin A_1_ (10 nM), concanamycin A (10 nM), AZM (50 *μ*g/ml), EM (50 *μ*g/ml), or CAM (50 *μ*g/ml) for various lengths of time. Cellular proteins were separated by 15% SDS-PAGE for LC3B and 11.25% SDS-PAGE for p62 and immunoblotted with anti-LC3B Ab and anti-p62 mAb. Immunoblotting with anti-GAPDH mAb was performed as an internal control. (B) U266 cells were cultured with AZM (50 *μ*g/ml) in the presence or absence of lysosomal inhibitors (LI), E-64d (10 *μ*g/ml) and pepstatin A (10 *μ*g/ml) for 24 h. Cellular proteins were separated by SDS-PAGE and immunoblotted as described above. The numbers indicate the ratios of LC3B-II/LC3B-I and p62/GAPDH as determined by densitometry.

We previously reported that combined treatment with BZ and bafilomycin A_1_, which is an autophagy inhibitor, synergistically enhanced ER-stress-mediated apoptosis in MM cells (10). Recent reports demonstrated that CAM attenuated the late stage of autophagy, although its mechanism still remains unclear (28). Additionally, AZM has been reported to block autophagy in macrophage (29). Since bafilomycin A_1_ is a macrolide, we speculated that macrolide antibiotics might share the same target(s) for blocking autophagy and might induce the same effect in MM cell growth. As indicated in Fig. 2A, immunoblotting with anti-LC3B Ab demonstrated that treatment of U266 cells with bafilomycin A_1_, concanamycin A, AZM, CAM or EM increased the expression ratios of LC3B-I to LC3B-II. However, p62, which is a substrate of autophagylysosomal proteolysis, increased after treatment with these macrolides. Unlike BZ, combined treatment with lysosomal inhibitors and AZM did not indicate any further increase of the LC3B-II/LC3B-I ratios, compared with those by treatment with lysosomal inhibitors or AZM alone (Fig. 2B). These data indicate that all macrolide antibiotics tested block autophagy flux.

**Figure 3:**
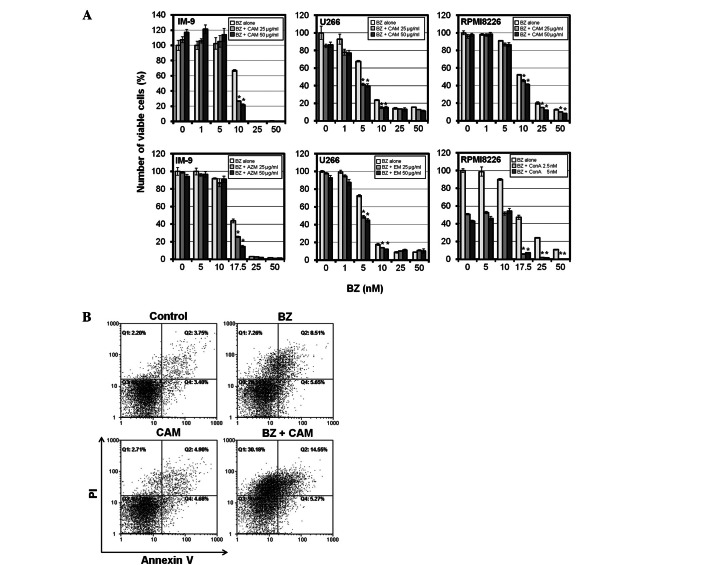
Combined treatment with BZ and macrolide antibiotics enhances cell growth inhibition and apoptosis in MM cell lines. (A) U266, IM-9 and RPMI8226 cells were cultured with BZ at various concentrations in the presence or absence of macrolide antibiotics [e.g., CAM, EM, AZM (at 25 or 50 *μ*g/ml) or concanamycin A (at 2.5 or 5 nM)] for 48 h. The number of viable cells was assessed as described in Materials and methods. ConA indicates concanamycin A. ^*^p<0.05, a macrolide antibiotic plus BZ vs. BZ/a macrolide antibiotic alone. (B) After IM-9 cells were treated with BZ (10 nM) and/or CAM (50 *μ*g/ml) for 16 h, apoptotic cells were assessed by flow cytometry as described in Materials and methods.

We next investigated whether a macrolide antibiotic increases the sensitivity of BZ in MM cells as well as bafilomycin A_1_(10). Treatment with AZM, CAM, or EM alone indicated little or almost no cytotoxicity at up to 100 *μ*g/ml in MM cell lines (data not shown). However, a combination of AZM, CAM, or EM (at 25 and 50 *μ*g/ml) with BZ enhanced BZ-induced cytotoxicity in MM cell lines including IM-9, U266 and RPMI8226 (Fig. 3A). In addition, flow cytometry of PI/Annexin V double staining revealed that CAM enhanced BZ-induced apoptosis in IM-9 cells, although treatment with CAM alone indicated no apoptosis induction (Fig. 3B).

### Accumulation of ubiquitinated proteins and aggresome formation after combined treatment with CAM plus BZ in MM cells

**Figure 4:**
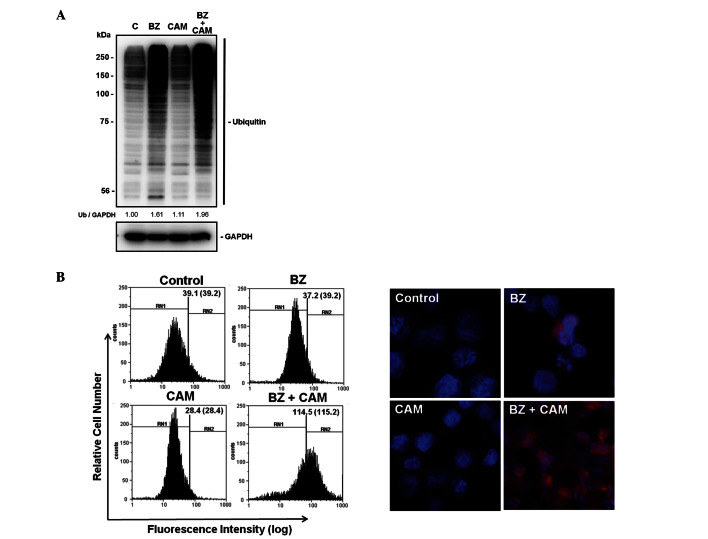
Cellular ubiquitinated proteins and aggresome formation after MM cell lines were treated with BZ and/or CAM. (A) After U266 cells were treated with BZ (5 nM) and/or CAM (50 *μ*g/ml) for 16 h, cellular proteins were lysed, separated by 11.25% SDS-PAGE and immunoblotted with anti-ubiquitin mAb. Immunoblotting with anti-GAPDH mAb was performed as an internal control. The numbers indicate the ratio of whole density of ubiquitinated proteins to GAPDH as determined by densitometry. (B) After IM-9 cells were treated with BZ (10 nM) and/or CAM (50 *μ*g/ml) for 16 h, aggresome formation was stained by a ProteoStat Protein Aggregation assay kit as described in Materials and methods. Aggresome (red) was detected by a fluorescence microscope (left panel). DAPI was used as a nuclear stain (blue). Alternatively, the fluorescence intensity of aggresome was assessed by flow cytometry (right panel). The number of each panel represents the fluorescence intensity of the mean channel number. The number in parenthesis represents the fluorescence peak intensity.

All data presented above suggest that two major intracellular proteolytic systems (e.g., the ubiquitin-proteasome system and the autophagy-lysosome system) could be simultaneously blocked by combined treatment with BZ and a macrolide antibiotic. It was reported that, in addition to proteasome-mediated protein degradation, polyubiquitinated proteins are also degraded by the autophagy-lysosome pathway via docking protein p62 which has both a ubiquitin-associated domain and an LC3-interacting lesion (25,26). Immunoblotting with anti-ubiquitin Ab indicate that treatment of U266 cells with BZ plus CAM further increased intracellular ubiquitinated proteins, compared with that by BZ treatment, while treatment with CAM alone had no effect on protein ubiquitination. Furthermore, aggresome formation was dramatically increased after combined treatment with BZ plus CAM in IM-9 cells (Fig. 4).

### Involvement of CHOP induction for enhanced cytotoxicity by combined treatment with BZ and CAM against MM cells

**Figure 5:**
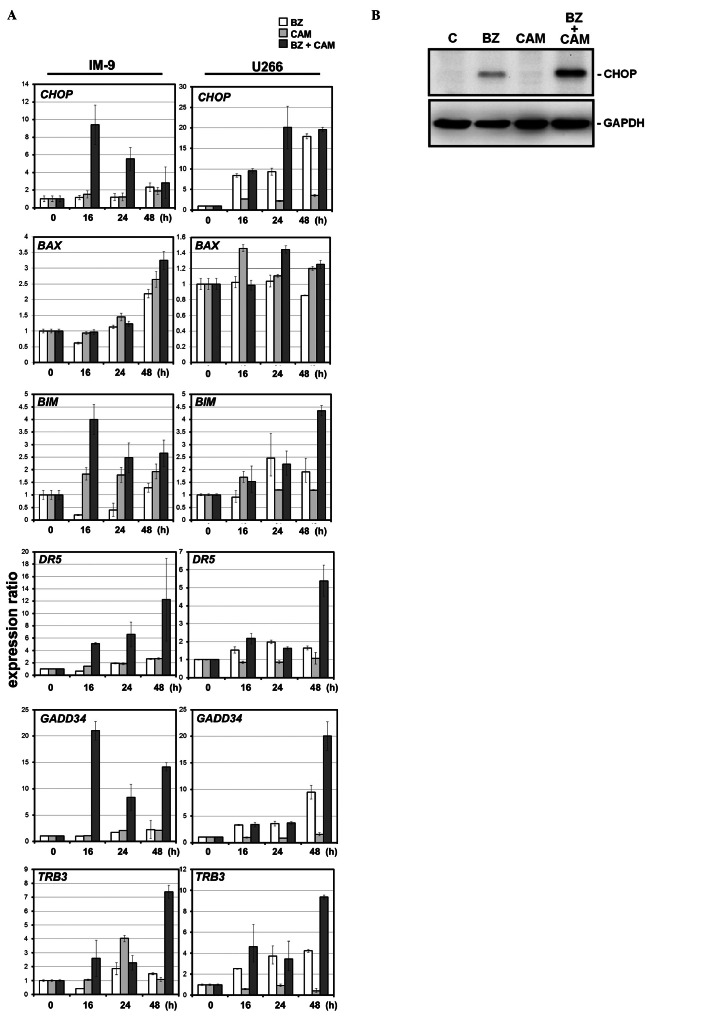
Profiles of the ER-stress-related genes and the target genes transcriptionally regulated by CHOP in IM-9 and U266 cells after treatment with BZ and/or CAM. (A) The expression of ER-stress-related genes in IM-9 and U266 cells, including CHOP and proapoptotic genes transcriptionally regulated by CHOP, were assessed by quantitative real-time PCR during 48-h exposure to BZ (10 nM for IM-9 cells, 5 nM for U266 cells), CAM (50 *μ*g/ml) and BZ+CAM. The data of the real-time PCR products for each gene were standardized to GAPDH as an internal control. The expression levels were compared with those in untreated cells. (B) Immunoblotting with anti-CHOP mAb after combined treatment of U266 cells with BZ and CAM. U266 cells were treated with/without CAM (50 *μ*g/ml) in the presence or absence of BZ (5 nM) for 24 h. Cellular proteins were separated by 11.25%, then immunoblotted with anti-CHOP mAb. Immunoblotting with anti-GAPDH mAb was performed as an internal control.

**Figure 6:**
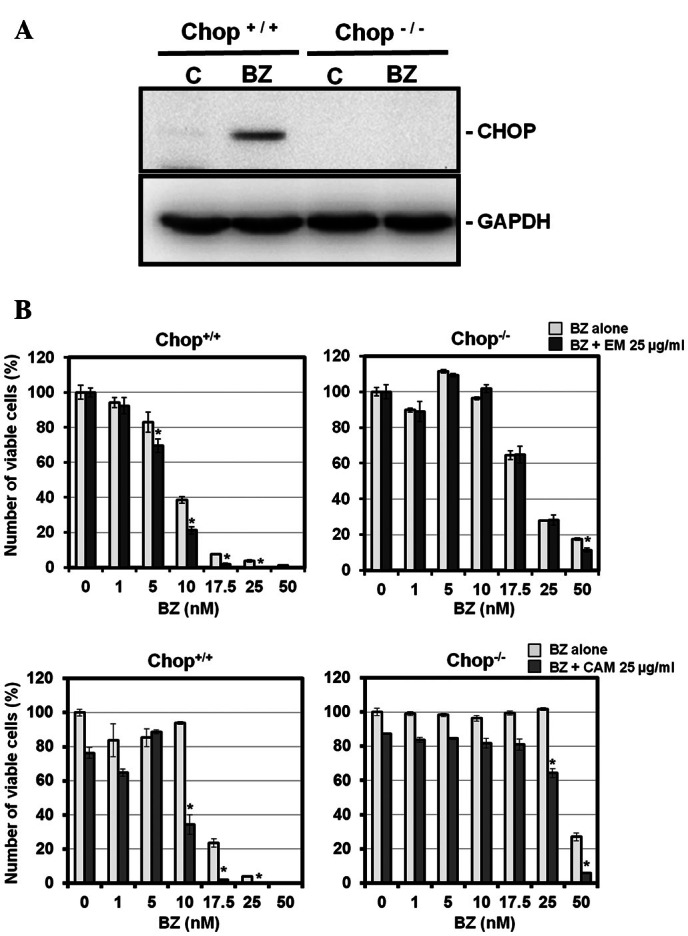
Cell growth inhibition of the CHOP^−/−^ MEF cell line and the wild-type MEF cell line after treatment with BZ and EM or CAM. (A) After treatment with BZ (10 nM) for 24 h, cellular proteins were separated by 11.25% SDS-PAGE and immunoblotted with anti-CHOP mAb. (B) The CHOP^−/−^ MEF cell line and the wild-type MEF cell line were cultured with BZ at various concentrations in the presence of absence of EM or CAM (at 25 *μ*g/ml) for 48 h. The number of viable cells was assessed by CellTiter Blue as described in Materials and methods. p<0.05, EM or CAM plus BZ vs. BZ/EM/CAM alone.

**Figure 7:**
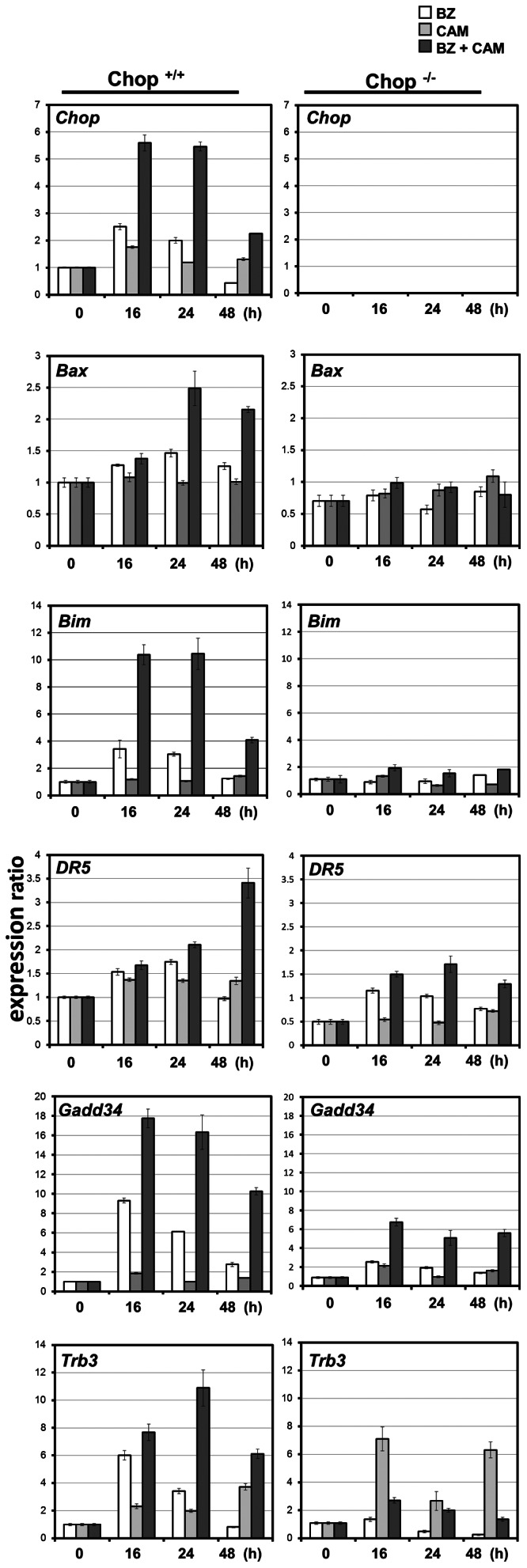
Expression profiles of ER-stress-related genes and the target genes transcriptionally regulated by CHOP after treatment with BZ and/or CAM in the wild-type MEF cell line and the CHOP^−/−^ MEF cell line. ER-stress related genes in the wild-type MEF cell line and the CHOP^−/−^ MEF cell line, including CHOP expressions, were assessed by quantitative real-time PCR during 48-h exposure to BZ (10 nM), CAM (50 *μ*g/ml) and BZ+CAM. The data of the real-time PCR products for each gene were standardized to GAPDH as an internal control. The expression levels were compared with those in untreated wild-type MEF cells.

It has been reported that ER-stress-mediated CHOP induction is involved in the cytotoxicity of BZ in various kinds of cells (10,33,34). This was also supported by data indicating that translational inhibition using cycloheximide attenuated BZ-induced cytotoxicity in U266 cells (data not shown). Therefore, we next examined whether combined treatment with BZ and CAM increases ER stress-loading on MM cells. Real-time PCR indicated that the levels of ER-stress-related genes were more pronounced by combined treatment with BZ and CAM than with either BZ or CAM alone (Fig. 5). Treatment with CAM alone indicated little effect on gene expression. In addition, proapoptotic genes (BIM, BAX, DR5 and TRB3) that are transcriptionally regulated by CHOP (35) were more pronounced with combined treatment than with treatment with BZ alone. These data strongly suggested that the simultaneous inhibition of two major protein degradation systems resulted in the enhancement of ER stress and appeared to lead to CHOP activation and subsequent apoptosis induction. To prove this hypothesis, we used a CHOP knockout MEF cell line. Fig. 6 illustrates that CHOP^−/−^ MEF cells were more resistant to BZ than wild-type MEF cells. Pronounced cytotoxicity was detected with combined treatment with BZ and EM or CAM in wild-type MEF cells as well as MM cell lines. It is noteworthy that this enhancement was almost completely canceled in CHOP^−/−^ MEF cells. This result indicates that cytotoxicity enhanced by a combination of BZ and EM or CAM is mediated through CHOP induction. Like MM cell lines, the expression profiles of CHOP-regulated proapoptotic genes were all pronounced with a combination of BZ and CAM in wild-type MEF cells, but not in CHOP^−/−^ MEF cells (Fig. 7).

## Discussion

**Figure 8:**
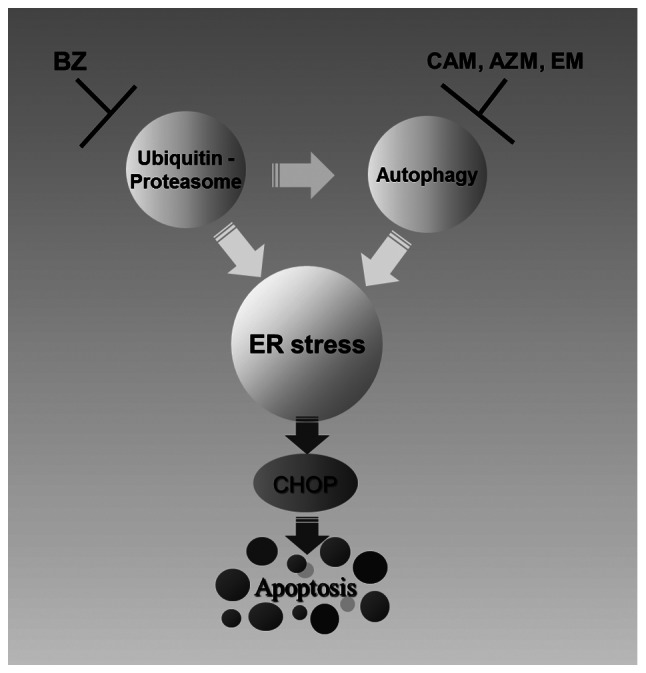
Induction of ER-stress-mediated apoptosis by inhibition of two major protein degradation systems in MM cells. Simultaneous inhibition of the ubiquitin-proteasome system by BZ and the autophagy-lysosome system by a macrolide antibiotic results in over-loading ER-stress on MM cells. This leads to activation of ER-stress-mediated apoptotic signals, including CHOP and subsequent upregulation of proapoptotic genes.

In the present study, we demonstrated that treatment with AZM, CAM, or EM, all of which are widely used macrolide antibiotics in routine medical care, enhanced BZ-induced cytotoxicity in MM cells, although these macrolides themselves exhibited almost no cytotoxicity (Fig. 3). Furthermore, we clearly demonstrated that combined treatment with BZ and one of the macrolides enhances CHOP induction and the expression levels of the proapoptotic genes transcriptionally regulated by CHOP (Figs. 5 and 7). Since CHOP knockout MEF cells completely canceled the enhanced cytotoxicity (Fig. 6), ER-stress-mediated CHOP induction appears to be involved in this phenomenon. In addition to the ubiquitinproteasome system, it was reported that polyubiquitinated proteins are engulfed into autophagosome and are degraded by the autophagy-lysosome system via binding to p62 docking protein, which has both an LC3-interacting region and a ubiquitin-associated domain (25,26). Thus, by binding ubiquitin via their C-terminal ubiquitin-associated domains, p62-mediated degradation of ubiquitinated cargo occurs by selective autophagy. First, we demonstrated that macrolide antibiotics suppressed autophagy flux, as previously reported with CAM and AZM (Fig. 2) (28,29). Therefore, blocking the two major protein degradation systems appears to result in loading excess ER stress, due to complete inhibition of ERAD (36,37). This was supported by our observation that intercellular ubiquitinated proteins were increased by BZ plus CAM, compared with that by BZ alone (Fig. 4A). Second, aggresome formation was dramatically increased by combining two reagents (Fig. 4B). Third, the expressions of ER-stress-related genes, including CHOP, were increased by combined treatment (Figs. 5 and 7). Therefore, simultaneous inhibition of the ubiquitin-proteasome system and the autophagy-lysosome system enhances ER-stress-mediated apoptosis in MM cells (Fig. 8).

A similar phenomenon was previously reported regarding enhanced cytotoxicity by the combination of BZ and an inhibitor for histone deacetylase 6 (HDAC6) (38). Unfolded proteins are transported to microtubule-organizing center (MTOC), where the lysosomes are enriched and degraded through the autophagy-lysosome pathway. HDAC6 deacetylates α-tubulin, which is thought to be a component of the MTOC; and knockdown of HDAC6 resulted in reducing autophagy (38). Tubacin, a small molecule inhibitor of HDAC6, prevented deacetylation of α-tubulin and produced accumulation of polyubiquitinated proteins and apoptosis and further acts synergistically with BZ to induce cytotoxicity in MM cells (39). Based on our results presented here, these data also can be explained by enhanced loading of ER-stress by simultaneously targeting the autophagy-lysosome pathway by tubacin and the ubiquitinprotease pathway by BZ (Fig. 8). In our system, dramatic enhancement of aggresome formation was detected (Fig. 4B). Aggresome formation therefore appears to provide another system for delivery of aggregated protein from cytoplasm to lysosomes for degradation and may reduce ER stress (40).

The molecular mechanism of autophagy induction in response to BZ is still unclear. A recent report demonstrated that BZ treatment induced autophagy in the breast cancer cell line MCF-7 by the proteasomal stabilization of ATF4 and ATF4-dependent upregulation of LC3B (41). ATF4, which is a transcription factor and a component of the PERK pathway in UPR (22), facilitated autophagy through direct binding to a cyclic AMP response element-binding site in the LC3B promoter, following upregulation of LC3B and autophagy induction in response to severe hypoxia (22,42,43). Therefore, crosstalk between the autophagy-lysosome system and ER stress was suggested (10,22). Although macrolides blocked the autophagy flux, combined treatment with BZ and one macrolide for >48 h resulted in enhanced autophagy induction, compared with BZ alone in U266 cells (data not shown).

The unexpected effects of macrolide antibiotics on MM cells discussed here are supported by several previous reports (28,29,44,45). CAM was reported to attenuate autophagy and to induce cell growth inhibition in MM cells (28), although our data indicated almost no cell growth inhibition. AZM also reportedly blocked autophagy in macrophage and was thus assumed to increase the risk of *Mycobacterium abcessu*s infection in cystic fibrosis patients who need to take AMZ for a long period (29). Furthermore, CAM enhanced tyrosine kinase inhibitor (TKI)-induced cell death in chronic myelogenous leukemia (CML) cells by inhibiting late-stage autophagy (44). Combining CAM plus TKI achieved remarkable molecular responses in four consecutive advanced-CML patients who were resistant to TKI alone (45). We have observed that CML cells are constitutively exposed to ER-stress with high expression levels of ER-stress-related genes, including GRP78 and CHOP, which may be due to abnormal BCR-ABL fusion protein synthesis (Miyazawa *et al*, unpublished data). Therefore, enhanced cytotoxicity could be explained in terms of loading excess ER-stress in CML cells. Interestingly, BiRD therapy consisting of Baixin (CAM), Revlimid (lenalidomide) and dexamethasone resulted in high complete and overall response rates in MM, although the molecular mechanism for using CAM was not clarified (46,47). CAM has immunomodulatory properties, partially mediated by the suppression of interleukin-6 and other inflammatory cytokines. There might also be a direct anti-neoplastic effect mediated by CAM (48). Certain macrolide antibiotics have been reported to exert some antitumor activities in non-small lung cancer and melanoma (49–52). Although the underlying molecular mechanism has not yet been clarified, ER-stress-mediated apoptosis might be involved in some of these antitumor activities.

It is also important to consider the target(s) of macrolides for blocking autophagy flux. Our previous report demonstrated that combined treatment with BZ and bafilomycin A_1_ (BAF), often used as an autophagy inhibitor, synergistically induces MM cell death *in vitro* (10). BAF is a macrolide antibiotic that was initially characterized for its selective inhibition of a proton-pumping V-ATPase (53). At nanomoler concentrations, BAF disrupts the vesicular proton gradients and ultimately increases the pH of acidic vesicles (53). This disruption of vesicular acidification in response to BAF appears to prevent the fusion of autophagosomes with lysosomes, resulting in inhibition of autophagy (54). It was reported that treatment with AZM increased lysosomal pH in macrophage, which may lead to inhibition of the lysosomal hydrolases having an optimal low pH for their enzymatic activities (29). Therefore, V-ATPase is a strong candidate for the target of macrolides. Further study is required to identify the target molecule(s) involved in this phenomenon.

Our study confirms that inhibiting the autophagy-lysosome system with a macrolide antibiotic can strongly enhance the efficacy of BZ and provides the foundation for clinical trials of BZ in combination with AZM, CAM, or EM for treating MM patients.

## Figures and Tables

**Table I:** Sequence of primers for real-time PCR.

Symbol	Species	Accession no.	Forward (5′-3′)	Reverse (5′-3′)	Products size (bp)
*CHOP*	h	NM_004083.5	AAATCAGAGCTGGAACCTGAGGA	CCATCTCTGCAGTTGGATCAGTC	112
	m	NM_007837.3	AATAACAGCCGGAACCTGAGGA	CCCAATTTCATCTGAGGACAGGA	200
*BAX*	h	NM_138761.3	GAACCATCATGGGCTGGACA	CCACAAAGATGGTCACGGTCTG	132
	m	NM_007527.3	CAGGATGCGTCCACCAAGAA	GTTGAAGTTGCCATCAGCAAACA	165
*BIM*	h	NM_207002.2	CATCATCGCGGTATTCGGTTC	AAGGTTGCTTTGCCATTTGGTC	141
	m	NM_207680.2	TCCTGTGCAATCCGTATCTCC	CGCAAGCTTCCATACGACAGT	70
*DR5*	h	NM_003842.4	AAGTGCCGCACAGGGTGTCC	GCTGGGACTTCCCCACTGTGC	116
	m	NM_020275.4	GTCCAGCTGGCCTACAGC	GCTTGCAGTTCCCTTCTGAC	87
*GADD34*	h	NM_014330.3	AACCAGCAGTTCCCTTCCTG	TTGCCTCTCGCTCACCATAC	74
	m	NM_008654.2	AGGAGAAGCTGGGTCCCTAC	GGTCACATCTTGGGTCAAGG	131
*TRB3*	h	NM_021158.3	CGCTGACCGTGAGAGGAAGAAGC	TCGGCTGCCTTGCCCGAGTA	159
	m	NM_175093.2	CGCTTTGTCTTCAGCAACTGT	TCATCTGATCCAGTCATCACG	83
*GAPDH*	h	NM_002046.3	GCACCGTCAAGGCTGAGAAC	TGGTGAAGACGCCAGTGGA	138
	m	NM_008084.2	TGTGTCCGTCGTGGATCTGA	TTGCTGTTGAAGTCGCAGGAG	150

h, human; m, mouse.
